# Supplementary material for: Circadian clock features define novel subtypes among breast cancer cells and shape drug sensitivity
Source: Mol Syst Biol. 2025 Feb 24;21(4):315–40. doi: 10.1038/s44320-025-00092-7 (PMC11965565; doi:10.1038/s44320-025-00092-7)
Supplement: Supplementary file 1 — Appendix [file 44320_2025_92_MOESM1_ESM.pdf]

Appendix

for

Circadian clock features define novel subtypes among breast cancer cell models and shape drug sensitivity

Authors: Carolin Ector[1-3#], Jeff Didier[4], Sébastien De Landtsheer[4], Malthe S. Nordentoft[5], Christoph Schmal[6], Ulrich Keilholz[1,7], Hanspeter Herzel[6,8], Achim Kramer[9], Thomas Sauter[4], Adrián E. Granada*[1,7]

Affiliations

1. Charité Comprehensive Cancer Center, Charité – Universitätsmedizin Berlin, 10117 Berlin, Germany.
2. BSIO Berlin School of Integrative Oncology, Charité – Universitätsmedizin Berlin, 10117 Berlin, Germany.
3. Faculty of Life Sciences, Humboldt-Universität zu Berlin, 10115 Berlin, Germany.
4. Department of Life Sciences and Medicine, University of Luxembourg, L-4365 Esch-sur-Alzette, Luxembourg.
5. Niels Bohr Institute, University of Copenhagen, 2100 Copenhagen, Denmark.
6. Institute for Theoretical Biology, Humboldt-Universität zu Berlin, 10115 Berlin, Germany.
7. German Cancer Consortium (DKTK), Berlin, Germany.
8. Charité – Universitätsmedizin Berlin, 10117 Berlin, Germany
9. Laboratory of Chronobiology, Charité – Universitätsmedizin Berlin, 10117 Berlin, Germany.

[#] **First author. * Corresponding author.** e-mail: adrian.granada@charite.de

Table of content

Appendix Figure S1. Continuous wavelet transform analysis. **2**

Appendix Figure S2. Capturing growth dynamics by long-term live-cell imaging. **3**

Appendix Figure S3. Elbow plot on selected circadian features from PCA clusters. **4**

Appendix Figure S4. Accuracy results of methods to assess the impact of circadian features on drug sensitivity.......... **5**

Appendix Figure S5. Accuracy results of classification methods to assess the impact of circadian features on drug sensitivity to multiple drugs. **6**

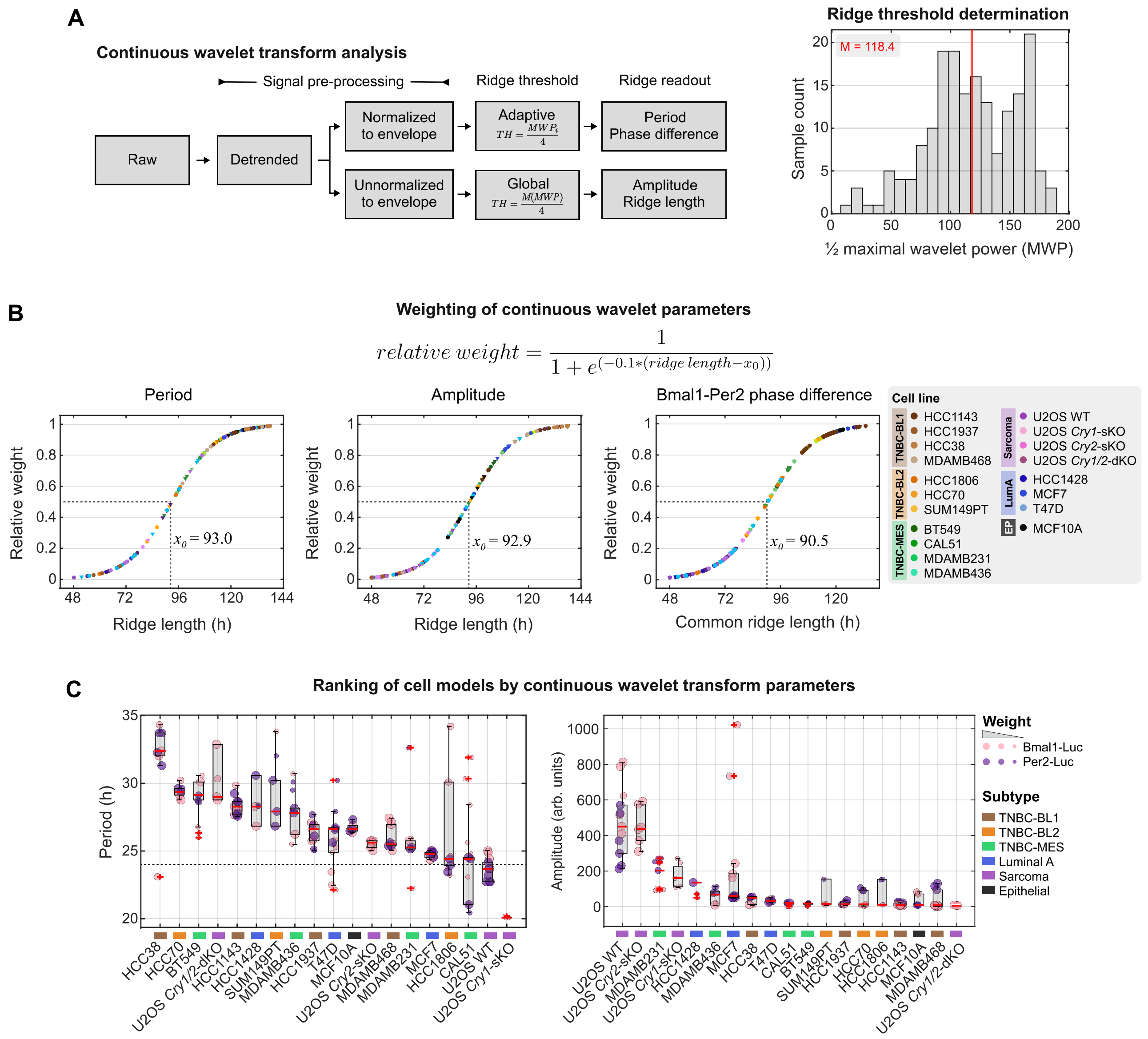

Appendix Figure S1. Continuous wavelet transform analysis. (**A**) Approach to extract ridge readout parameters from wavelet spectra (left panel). Ridge thresholds (TH) applied to the power spectrum were either adaptive, set to a quarter of the sample's maximum wavelet power (MWP), or global, set to a quarter of the median of the maximum wavelet power of all samples combined (right panel, n=180 samples). (**B**) Calculation of relative weights of continuous wavelet transform (CWT) parameters through sigmoidal fitting of ridge lengths underlying the extracted periods (left panel) and amplitudes (middle panel); weighting of *Bmal1-Per2* phase differences were based on the common ridge lengths of *Bmal1-* and *Per2*-phases (right panel). Cell line models are color-coded by their subtype. The hill coefficient of the sigmoidal model was set to -0.1 and calculated inflection points (x_0) of the fitted curve are marked in the plots. (**C**) Weighted boxplot of median periods (left panel) and amplitudes (right panel) from *Bmal1-* and *Per2*-signals of the specified cell models, with the bottom and top edges of the boxes representing the 25th and 75th percentiles, respectively. Extending whiskers represent data points within 1.5 times the interquartile range from lower and upper quartile. Relative weights of the samples, calculated as shown in B, are indicated by datapoint sizes: small (≤ 0.33), intermediate (> 0.33 and ≤ 0.66), and large (> 0.66). Red horizontal lines denote median values, red crosses mark outliers

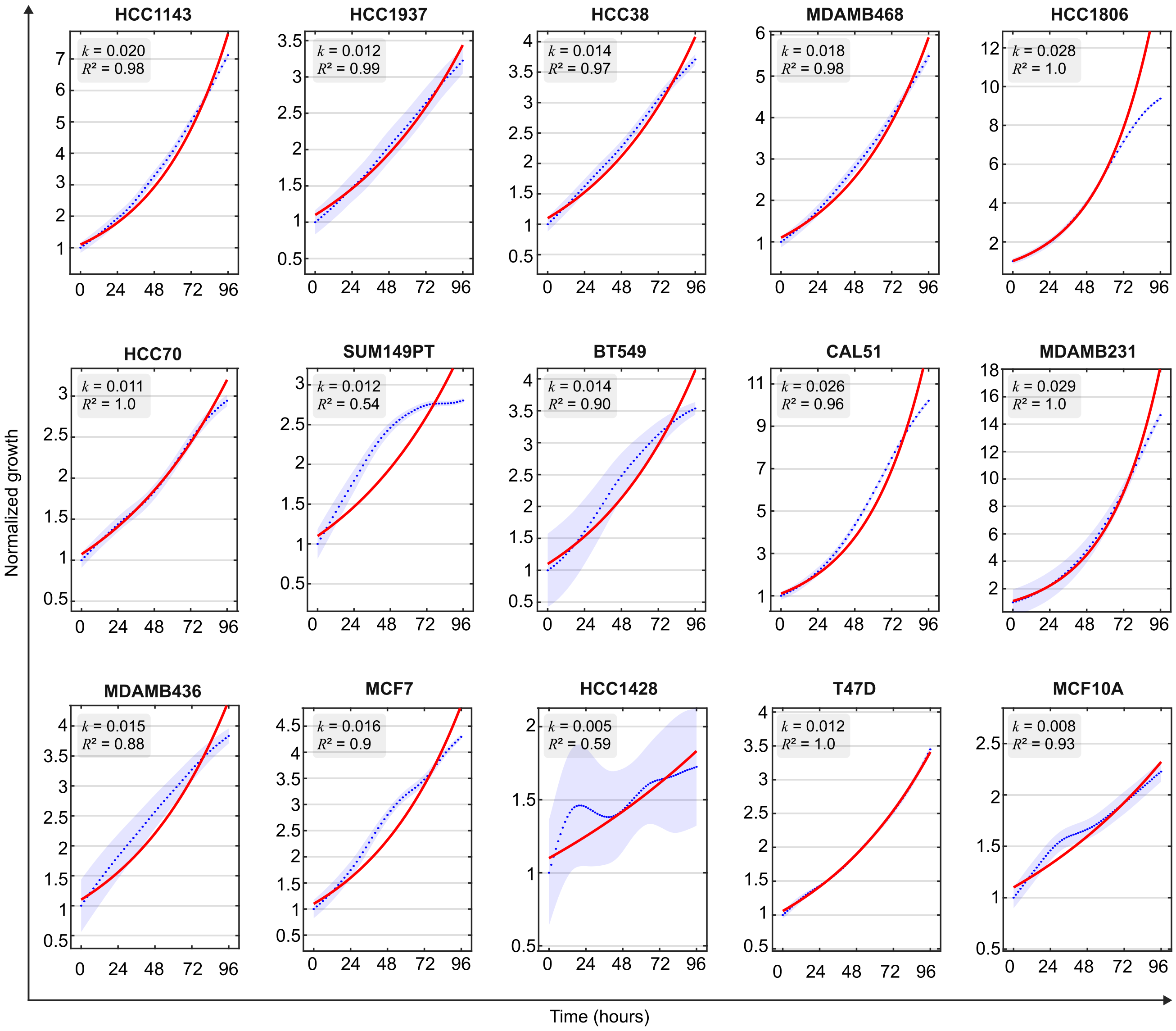

Appendix Figure S2. Capturing growth dynamics by long-term live-cell imaging. Normalized growth curves (blue dots) and exponential fits (red lines) for the indicated cell line models, yielding growth rates (k), and fit accuracies (R^2). Growth assessment was based on nucleus counts, except for BT549 and MDAMB436, where confluency was used. Data represents the mean±s.d. of 9 images taken in a single well, with the shaded area representing the standard deviation across the images.

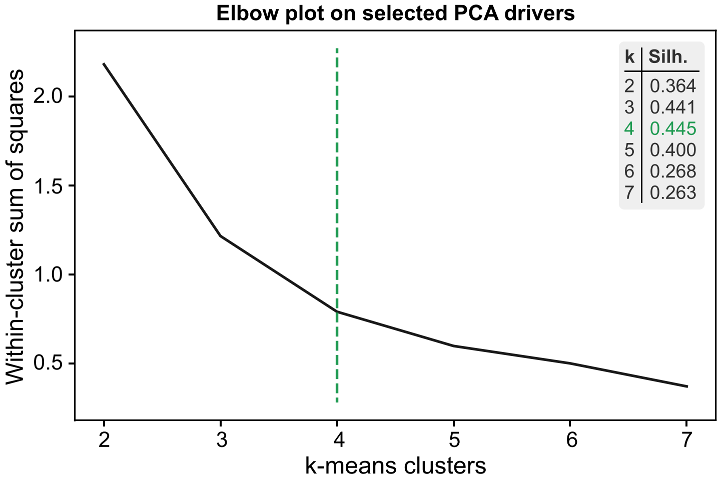

Appendix Figure S3. Elbow plot on selected circadian features from PCA clusters. Within-cluster sum of squares of each k-means cluster, clustering 15 cell lines by their circadian signal's period, circadian component, and *Bmal1-Per2* phase difference. The green dashed line marks the elbow point, representing the optimal number of circadian-based clusters (k) within the cell line panel. Cluster-specific silhouette (Silh.) scores are denoted in the grey box.

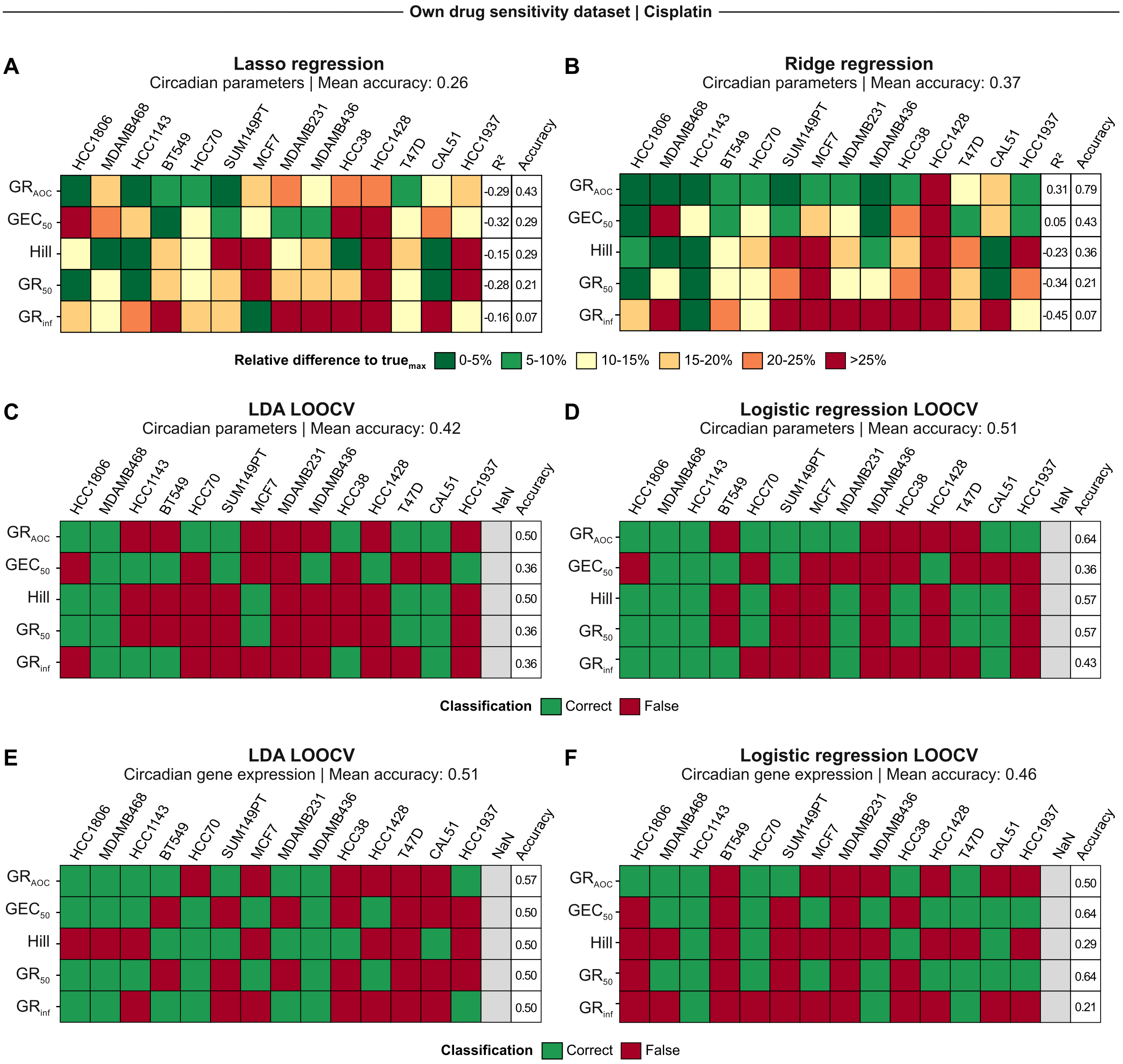

Appendix Figure S4. Accuracy results of methods to assess the impact of circadian features on drug sensitivity. (**A**) Accuracy of lasso regression in predicting cisplatin sensitivity metrics (rows) from circadian parameters across cell line models (columns). Accuracy was measured as the normalized difference between true and predicted sensitivity values per cell models (color-coded) and averaged per row. Predictions within 10% deviation were deemed correct. Mean accuracies across all sensitivity parameters are stated in the heading. R^2 values of the fit are provided alongside accuracy. (**B**) See A, but shown for lasso regression. (**C**) Leave-one-out cross-validation (LOOCV) of LDA results assessing the ability of circadian parameters to distinguish between high and low sensitivity groups to cisplatin (based on median-binarized drug sensitivity-values). The color-coding indicates classification accuracy for each cell line model: green for correct and red for incorrect categorization. The rightmost column shows total LOOCV accuracy for each sensitivity parameter, with 1 indicating 100% accuracy. Mean accuracies across all sensitivity parameters are stated in the heading. (**D**) See C, but shown for logistic regression. (**E**) See C, but shown for LDA on circadian gene expression values. (**F**) See C, but shown for logistic regression on circadian gene expression values.

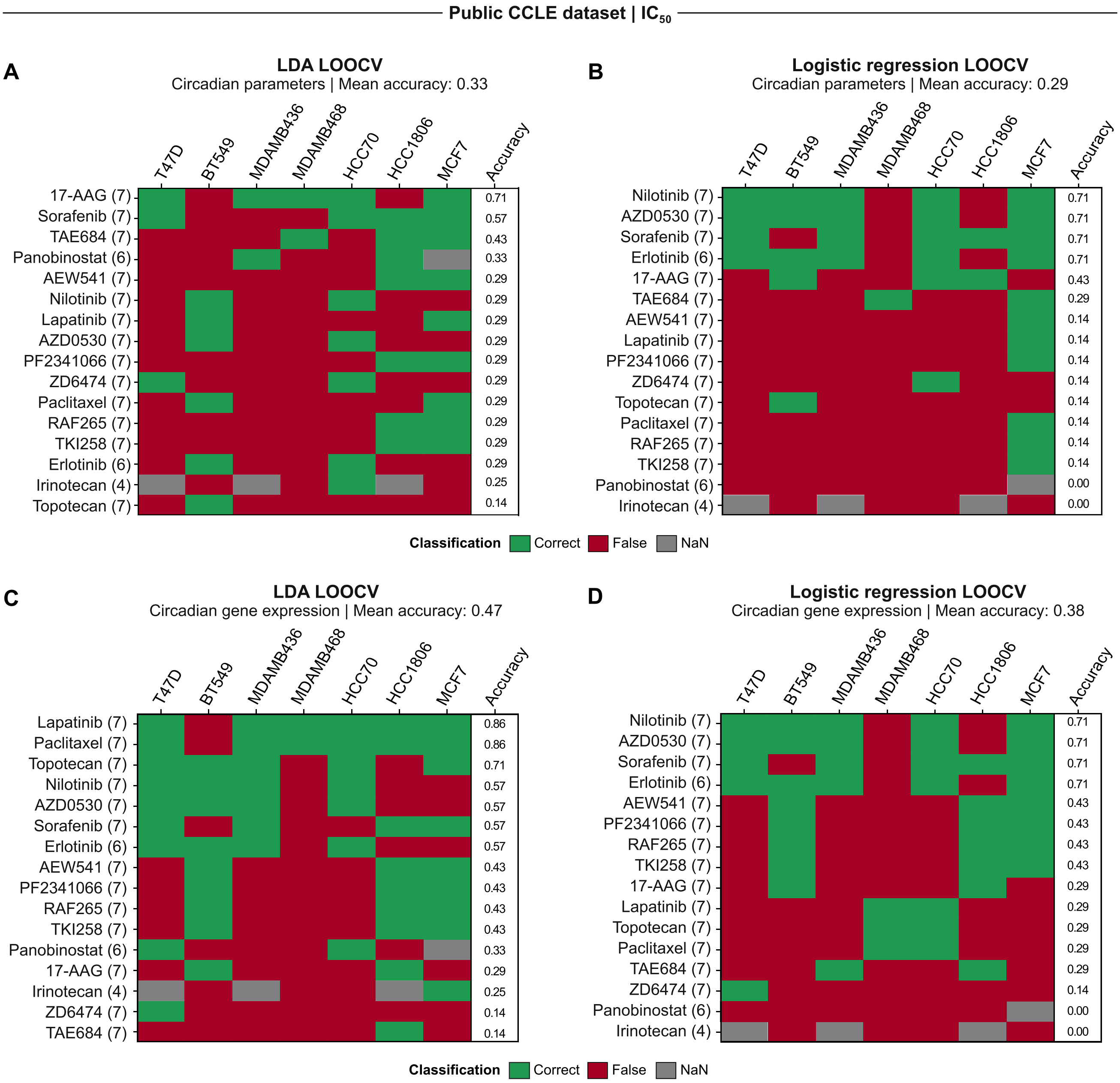

Appendix Figure S5. Accuracy results of classification methods to assess the impact of circadian features on drug sensitivity to multiple drugs. (**A**) Leave-one-out cross-validation (LOOCV) of LDA results assessing the ability of circadian parameters to distinguish between high and low sensitivity groups to numerous drugs from the CCLE database (based on median-binarized IC_{50} values). The color-coding indicates classification accuracy for each cell line model: green for correct and red for incorrect categorization. The rightmost column shows total LOOCV accuracy for each sensitivity parameter, with 1 indicating 100% accuracy. Mean accuracies across all drugs are stated in the heading. NaN, cell line not available. (**B**) See A, but shown for logistic regression. (**C**) See A, but shown for LDA on circadian gene expression values. (**D**) See A, but shown for logistic regression on circadian gene expression values.
