# Supplementary material for: Circadian clock features define novel subtypes among breast cancer cells and shape drug sensitivity
Source: Mol Syst Biol. 2025 Feb 24;21(4):315–40. doi: 10.1038/s44320-025-00092-7 (PMC11965565; doi:10.1038/s44320-025-00092-7)
Supplement: Supplementary file 8 — Expanded View Figures [file 44320_2025_92_MOESM8_ESM.pdf]

Expanded View Figures

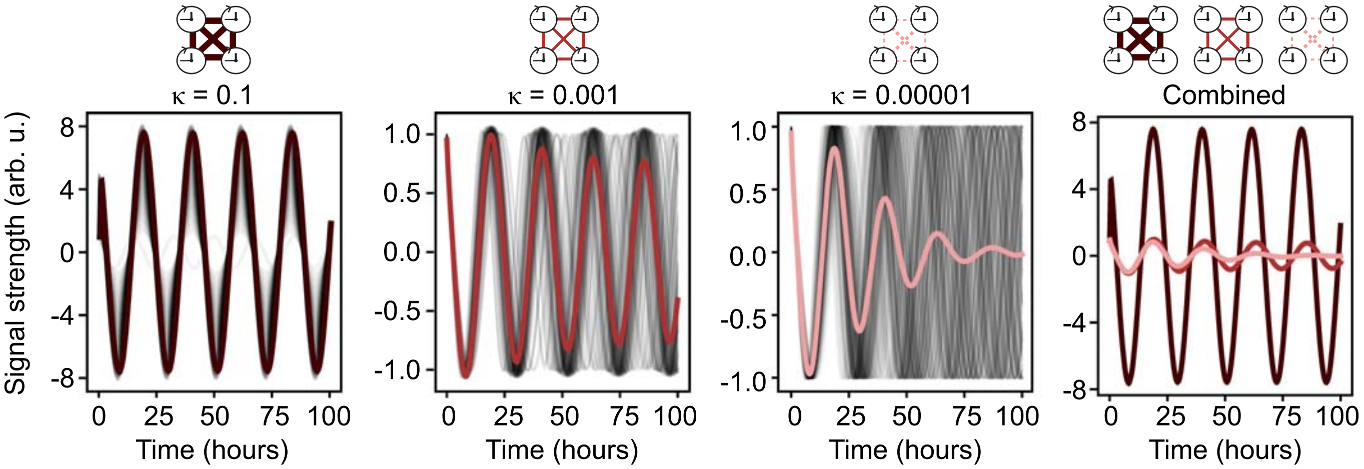

Figure EV1. Expanded view for Fig. 2.

Simulated oscillators of varying coupling strengths κ. Individual traces of each oscillator are shown in black, collective signals are shown as thick lines with gradients of red, based on their coupling strength, where dark red denotes high coupling strength, and pink refers to low coupling. The right panel is a composite of all collective signals.

 © The Author(s)

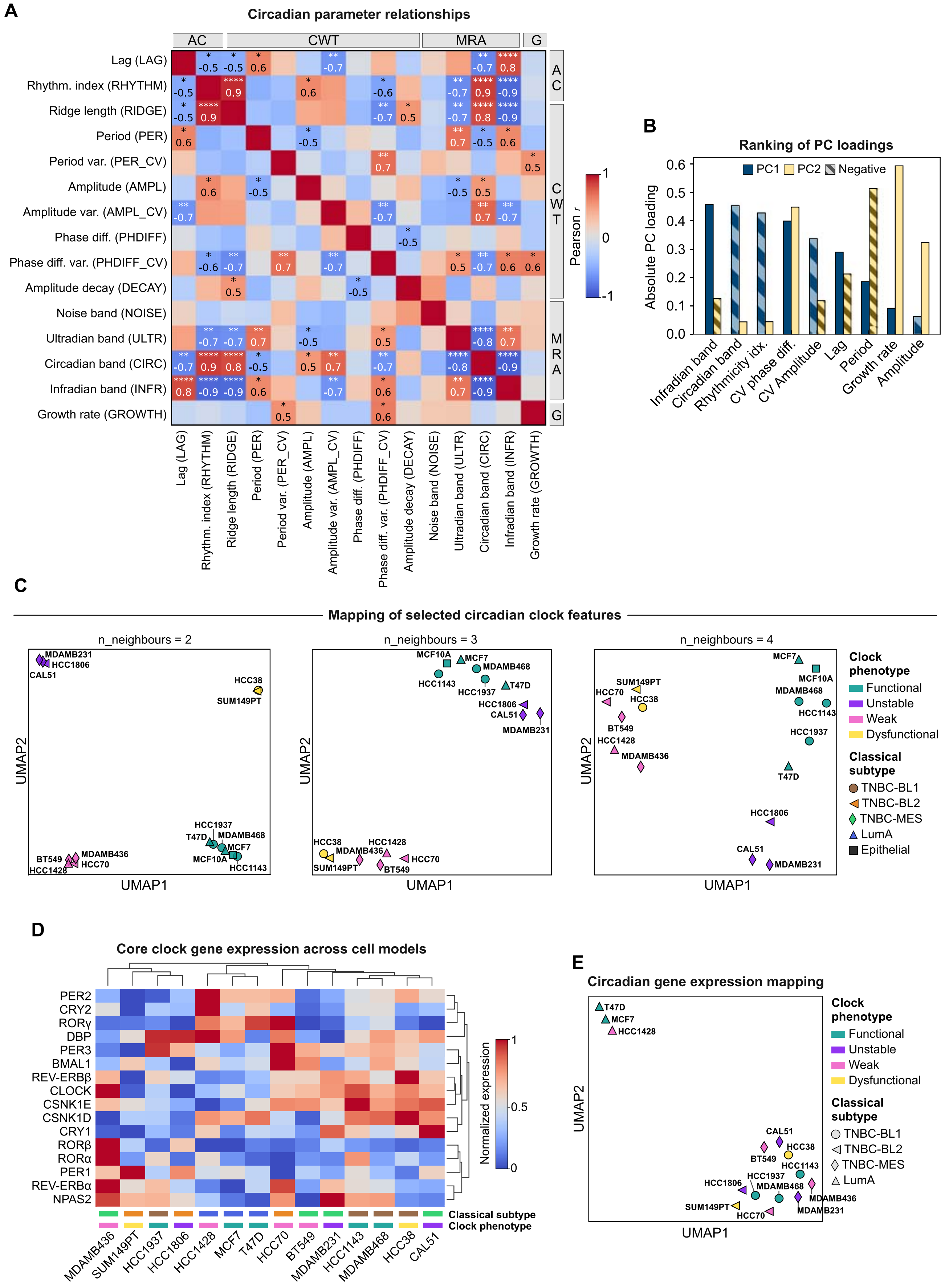
A
Circadian parameter relationships
AC
CWT
MRA
G
Lag (LAG)
Rhythm. index (RHYTHM)
Ridge length (RIDGE)
Period (PER)
Period var. (PER_CV)
Amplitude (AMPL)
Amplitude var. (AMPL_CV)
Phase diff. (PHDIFF)
Phase diff. var. (PHDIFF_CV)
Amplitude decay (DECAY)
Noise band (NOISE)
Ultradian band (ULTR)
Circadian band (CIRC)
Infradian band (INFR)
Growth rate (GROWTH)
Pearson r
B
Ranking of PC loadings
PC1
PC2
Negative
Absolute PC loading
Infradian band
Circadian band
Rhythmicity idx.
CV phase diff.
CV Amplitude
Lag
Period
Growth rate
Amplitude
C
Mapping of selected circadian clock features
n_neighbours = 2
n_neighbours = 3
n_neighbours = 4
UMAP1
UMAP2
Clock phenotype
Functional
Unstable
Weak
Dysfunctional
Classical subtype
TNBC-BL1
TNBC-BL2
TNBC-MES
LumA
Epithelial
D
Core clock gene expression across cell models
PER2
CRY2
RORγ
DBP
PER3
BMAL1
REV-ERBβ
CLOCK
CSNK1E
CSNK1D
CRY1
RORβ
RORα
PER1
REV-ERBα
NPAS2
Normalized expression
Classical subtype
Clock phenotype
MDAMB436
SUM149PT
HCC1937
HCC1806
HCC1428
MCF7
T47D
HCC70
BT549
MDAMB231
HCC1143
MDAMB468
HCC38
CAL51
E
Circadian gene expression mapping
UMAP1
UMAP2
Clock phenotype
Functional
Unstable
Weak
Dysfunctional
Classical subtype
TNBC-BL1
TNBC-BL2
TNBC-MES
LumA

Figure EV2. Expanded view for Fig. 3.

(**A**) Pearson correlation coefficients between the complete set of *Bmal1-Per2*-averaged circadian features and growth rates across all breast cancer cell models and the epithelial MCF10A cell line ($n = 15$ cell lines). Parameters are categorized by their approach of calculation (refer to Fig. 3A legend). Displayed are statistically significant correlation values, where *, **, and **** indicate p-values < 0.05, 0.01, and 0.0001, respectively. Exact p-values: RIDGE-LAG = 4.2×10^{-2}; RIDGE-RHYTHM = 2.8×10^{-7}; PHDIFF_RIDGE = 2×10^{-3}; PHDIFF_CV-PER_CV = 3×10^{-3}; DECAY-RIDGE = 4.3×10^{-2}; DECAY-PHDIFF = 3.3×10^{-2}; ULTR-RHYTHM = 3×10^{-3}; ULTR-RIDGE = 4×10^{-3}; ULTR-PER = 3×10^{-3}; ULTR-AMPL = 3.8×10^{-2}; ULTR-PHDIFF_CV = 4.1×10^{-2}; ULTR-INFR = 5.5×10^{-3}; CIRC-RIDGE = 8×10^{-5}; CIRC-ULTR = 8.8×10^{-5}; INFR-RIDGE = 1.8×10^{-5}; INFR-ULTR = 5.5×10^{-3}; INFR-CIRC = 1×10^{-6}; GROWTH-PER_CV = 3.7×10^{-2}. Refer to Fig. 3A for all other significant p-values. (**B**) Ranking of absolute PC loadings for each circadian and growth parameter, corresponding to the PCA biplot shown in Fig. 3B. Parameters are sorted in descending order based on their absolute contribution in the first principal component. Negative values are shaded. (**C**) Uniform Manifold Approximation and Projection (UMAP) of cell models complementary to the k-means clustering analysis in Fig. 3C. The classical subtype and clock phenotype of each model is illustrated by different markers and colors, respectively. Nearest neighbors = 2–4. (**D**) Cluster map of core clock gene expression values across breast cancer cell models, using the Euclidian distance method. Color-coded rectangles above the cell line names indicate the classical subtype and clock phenotype. Refer to (**C**) for color-coding. (**E**) UMAP of cell models based on core circadian gene expression values shown in (**D**). The classical subtype and clock phenotype of each model is illustrated by different markers and colors, respectively. Nearest neighbors = 3.

 © The Author(s)

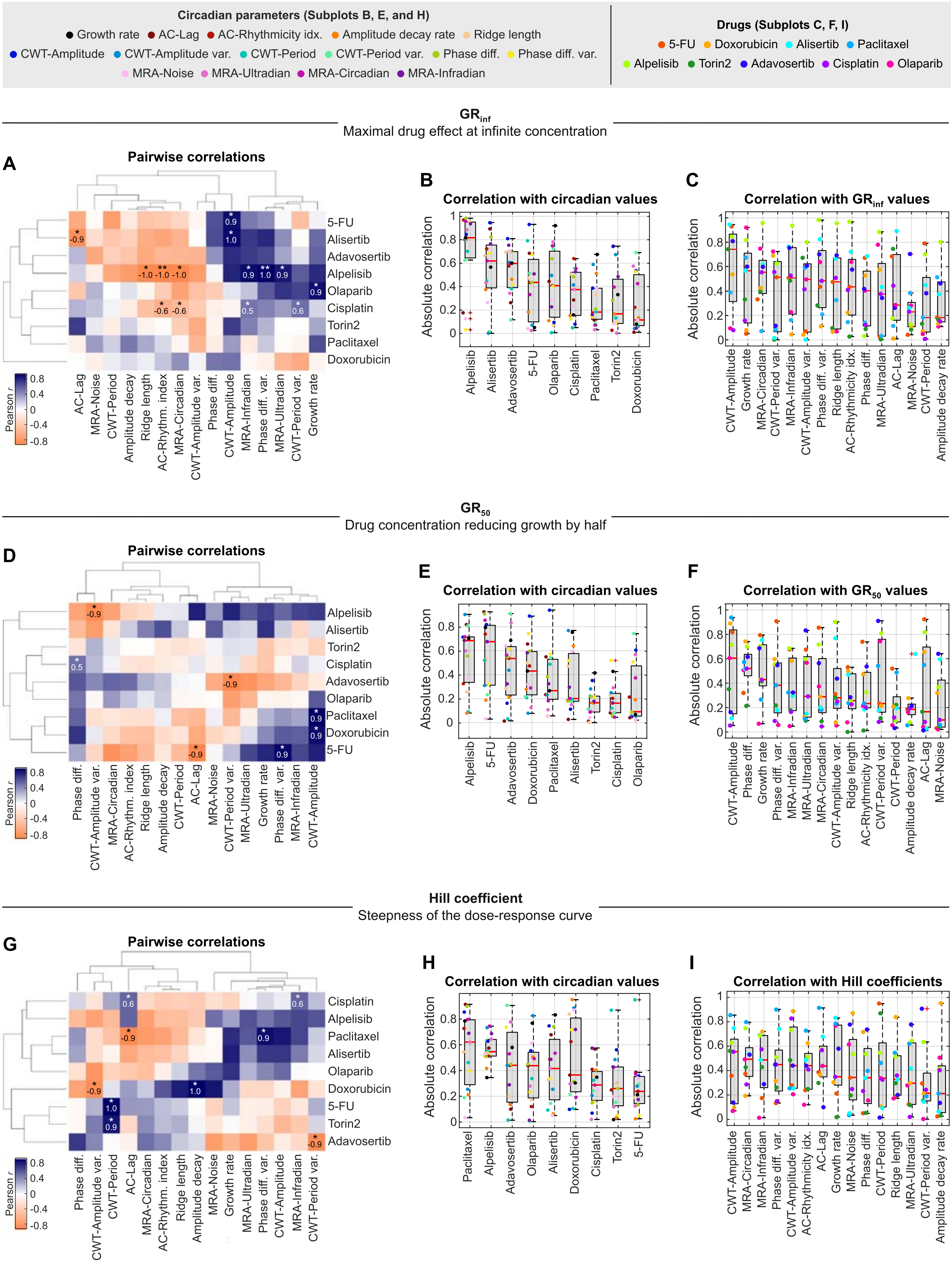
Circadian parameters (Subplots B, E, and H)
Growth rate
AC-Lag
AC-Rhythmicity idx.
Amplitude decay rate
Ridge length
CWT-Amplitude
CWT-Amplitude var.
CWT-Period
CWT-Period var.
Phase diff.
Phase diff. var.
MRA-Noise
MRA-Ultradian
MRA-Circadian
MRA-Infradian
Drugs (Subplots C, F, I)
5-FU
Doxorubicin
Alisertib
Paclitaxel
Alpelisib
Torin2
Adavosertib
Cisplatin
Olaparib
GRinf
Maximal drug effect at infinite concentration
A
Pairwise correlations
5-FU
Alisertib
Adavosertib
Alpelisib
Olaparib
Cisplatin
Torin2
Paclitaxel
Doxorubicin
AC-Lag
MRA-Noise
CWT-Period
Amplitude decay
Ridge length
AC-Rhythm. index
MRA-Circadian
CWT-Amplitude var.
Phase diff.
CWT-Amplitude
MRA-Infradian
Phase diff. var.
MRA-Ultradian
CWT-Period var.
Growth rate
Pearson r
B
Correlation with circadian values
Absolute correlation
C
Correlation with GRinf values
GR50
Drug concentration reducing growth by half
D
Pairwise correlations
E
Correlation with circadian values
F
Correlation with GR50 values
Hill coefficient
Steepness of the dose-response curve
G
Pairwise correlations
H
Correlation with circadian values
I
Correlation with Hill coefficients

© The Author(s)

Figure EV3. Expanded view for Fig. 4.

(**A**) Hierarchical clustering of Pearson correlation coefficients between GR_{inf} values for 9 drugs (rows) and 15 circadian clock and growth parameters (columns, averaged *Bmal1-Per2* data). Shown are statistically significant correlation values, where *, and **, indicate *p*-values 0.05, and 0.01, respectively. exact *p*-values: 5-FU-Amplitude = 2.2×10^{-2}; Alisertib-Lag = 4.1×10^{-2}; Alisertib-Amplitude = 1.5×10^{-2}; Alpelisib-Ridge = 1×10^{-2}; Alpelisib-Rhythmicity = 6.6×10^{-3}; Alpelisib-Circadian = 1×10^{-2}; Alpelisib-Infradian = 1.9×10^{-2}; Alpelisib-Phase diff. var = 2.7×10^{-3}; Alpelisib-Ultradian = 4.6×10^{-2}; Olaparib-Growth = 2.7×10^{-2}; Cisplatin-Rhythmicity = 1×10^{-2}; Cisplatin-Circadian = 3.2×10^{-2}; Cisplatin-Infradian = 4.4×10^{-2}; Cisplatin-Period var. = 3.1×10^{-2}. $n = 5$ cell lines per drug, except for cisplatin, where $n = 15$ cell lines. (**B**) Ranking of absolute correlation values between cellular GR_{inf} values and circadian clock/growth parameters, accumulated by individual drugs ($n = 15$ parameters). Bottom and top edges of the boxes represent the 25th and 75th percentiles, respectively. Extending whiskers represent data points within 1.5 times the interquartile range from lower and upper quartile. Red horizontal lines denote median values, red crosses mark outliers. (**C**) Ranking of absolute correlation values between cellular GR_{inf} values and circadian clock/growth parameters, accumulated by individual parameter ($n = 9$ drugs). See (**B**) for definition of boxes. (**D**) See (**A**), but shown for GR_{50} values. *p*-values: Alpelisib-Amplitude var. = 3.6×10^{-2}; Cisplatin-Phase diff = 4.8×10^{-2}; Adavosertib-Period var. = 3.2×10^{-2}; Paclitaxel-Amplitude = 1.8×10^{-2}; Doxorubicin-Amplitude = 4.4×10^{-2}; 5-FU-Lag = 2.5×10^{-2}; 5-FU=Phase diff. var. = 3.3×10^{-2}. (**E**) See (**B**), but shown for GR_{50} values. (**F**) See (**C**), but shown for GR_{50} values. (**G**) See (**A**), but shown for Hill coefficient values. *p*-values: Cisplatin-Lag = 2.7×10^{-2}; Cisplatin-Infradian = 2.5×10^{-2}; Paclitaxel-Lag = 3×10^{-2}; Paclitaxel-Phase diff. var. = 3.7×10^{-2}; Doxorubicin-Amplitude var. = 4.5×10^{-2}; Doxorubicin-Amplitude decay = 1.2×10^{-2}; 5-FU-Period = 1.3×10^{-2}; Torin2-Period = 2.4×10^{-2}; Adavosertib-Period var. = 3.4×10^{-2}; (**H**) See (**B**), but shown for Hill coefficient values. (**I**) See (**C**), but shown for Hill coefficient values.

 © The Author(s)

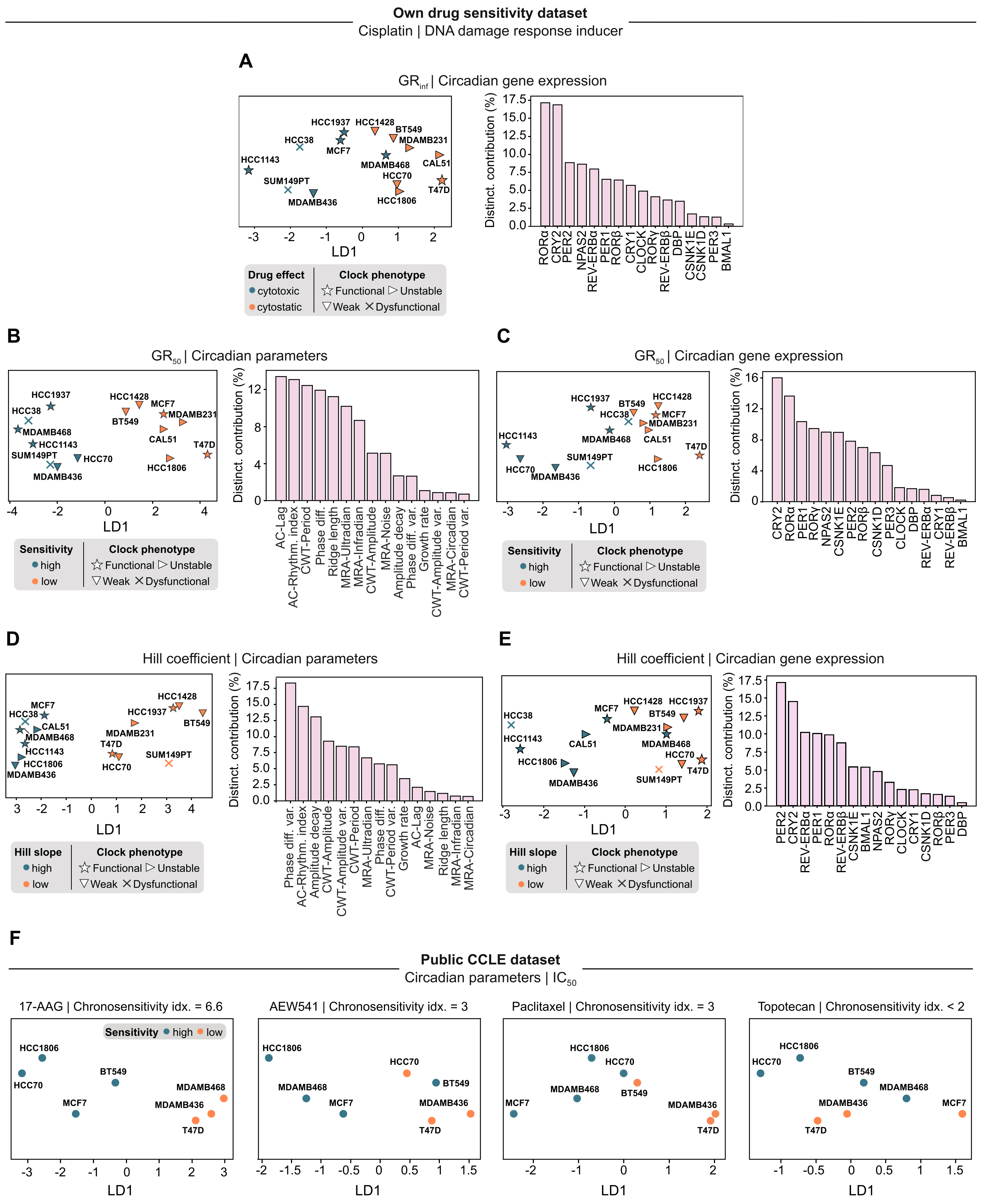
Own drug sensitivity dataset
Cisplatin | DNA damage response inducer
A
GRinf | Circadian gene expression
HCC1937 HCC1428 BT549 MDAMB231 HCC38 MCF7 HCC1143 MDAMB468 CAL51 HCC70 SUM149PT T47D MDAMB436 HCC1806
LD1
Distinct. contribution (%)
RORα CRY2 PER2 NPAS2 REV-ERBα PER1 RORβ CRY1 CLOCK RORγ REV-ERBβ DBP CSNK1E CSNK1D PER3 BMAL1
Drug effect: cytotoxic, cytostatic
Clock phenotype: Functional, Unstable, Weak, Dysfunctional
B
GR50 | Circadian parameters
HCC1937 HCC1428 MCF7 HCC38 MDAMB231 BT549 MDAMB468 HCC1143 CAL51 T47D SUM149PT HCC70 HCC1806 MDAMB436
LD1
Distinct. contribution (%)
AC-Lag AC-Rhythm. index CWT-Period Phase diff. Ridge length MRA-Ultradian MRA-Infradian CWT-Amplitude MRA-Noise Amplitude decay Phase diff. var. Growth rate CWT-Amplitude var. MRA-Circadian CWT-Period var.
Sensitivity: high, low
Clock phenotype: Functional, Unstable, Weak, Dysfunctional
C
GR50 | Circadian gene expression
HCC1937 BT549 HCC1428 HCC38 MCF7 MDAMB231 HCC1143 MDAMB468 CAL51 T47D SUM149PT HCC70 MDAMB436 HCC1806
LD1
Distinct. contribution (%)
CRY2 RORα PER1 RORγ NPAS2 CSNK1E PER2 RORβ CSNK1D PER3 CLOCK DBP REV-ERBα CRY1 REV-ERBβ BMAL1
Sensitivity: high, low
Clock phenotype: Functional, Unstable, Weak, Dysfunctional
D
Hill coefficient | Circadian parameters
HCC1428 MCF7 HCC1937 HCC38 BT549 CAL51 MDAMB231 MDAMB468 T47D HCC1143 SUM149PT HCC1806 HCC70 MDAMB436
LD1
Distinct. contribution (%)
Phase diff. var. AC-Rhythm. index Amplitude decay CWT-Amplitude CWT-Amplitude var. CWT-Period MRA-Ultradian Phase diff. CWT-Period var. Growth rate AC-Lag MRA-Noise Ridge length MRA-Infradian MRA-Circadian
Hill slope: high, low
Clock phenotype: Functional, Unstable, Weak, Dysfunctional
E
Hill coefficient | Circadian gene expression
HCC38 MCF7 HCC1428 HCC1937 BT549 MDAMB231 HCC1143 CAL51 MDAMB468 HCC70 HCC1806 T47D MDAMB436 SUM149PT
LD1
Distinct. contribution (%)
PER2 CRY2 REV-ERBα PER1 RORα REV-ERBβ CSNK1E BMAL1 NPAS2 RORγ CLOCK CRY1 CSNK1D RORβ PER3 DBP
Hill slope: high, low
Clock phenotype: Functional, Unstable, Weak, Dysfunctional
F
Public CCLE dataset
Circadian parameters | IC50
17-AAG | Chronosensitivity idx. = 6.6
AEW541 | Chronosensitivity idx. = 3
Paclitaxel | Chronosensitivity idx. = 3
Topotecan | Chronosensitivity idx. < 2
Sensitivity: high, low
HCC1806 HCC70 BT549 MDAMB468 MCF7 MDAMB436 T47D
LD1

Figure EV4. Expanded view for Fig. 4.

(**A**) Linear discriminant analysis (LDA) on median-binarized cisplatin GR_{inf} values using circadian gene expression data as input. Cell models shown in with GR_{inf} values below or above the median are colored in blue-green and orange, respectively. The right bar plots rank the individual contribution of each input parameter to the obtained discriminative information. (**B**) See (**A**), but shown for GR_{50} values and *Bmal1-Per2* oscillation and growth parameters as input. (**C**) See (**A**), but shown for GR_{50} values. (**D**) See (**B**), but shown for Hill coefficient values and *Bmal1-Per2* oscillation and growth parameters as input. (**E**) See (**A**), but shown for Hill coefficient values. (**F**) LDA profiles of different drugs, exemplifying cell model distributions along LD1 for varying chronosensitivity indices, sorted from highest index (left panel) to lowest (right panel).

 © The Author(s)
